# A case of pleural effusion caused by infection from *Toxocara canis*

**DOI:** 10.1186/1939-4551-8-S1-A131

**Published:** 2015-04-08

**Authors:** Rosanna Qualizza, Cristoforo Incorvaia, Policlinico Anna Maraschini

**Affiliations:** 1 Istituti Clinici Di Perfezionamento, Italy; 2 Irccs Fondazione Ca' Granda Ospedale Maggiore, Italy

## Background

*Toxocara canis* is an intestinal nematode affecting dogs and cats which causes human infestation through the ingestion of embryonated eggs excreted in faeces. Once larvae have migrated to various tissues and organs, they can cause a wide array of clinical symptoms. We describe a case of pleural effusion caused by *T. canis* infection.

## Methods

The patient was a 56-year old Caucasian woman suffering from rheumatoid arthritis since 1995. She was subsequently diagnosed with Sjoegren’s syndrome and autoimmune thyroiditis. In 2009, the patient had a skin rash which disappeared after corticosteroid treatment. In January 2012 a routine chest X-ray detected a pleural effusion, that was treated by various cycles of antibiotics and corticosteroids without improvement. The patient was then referred to us because of a concomitant eosinophilia. She also had difficulty in breathing, and allergy was suspected as a possible cause. The patient underwent allergy tests, parasitological evaluation and a routine blood examination, including IgG antibodies to *T. canis.*

## Results

Allergy tests were negative, while IgG antibodies to T. canis were positive by both ELISA and Western Blotting. An anti-elminthic treatment was prescribed using mebendazole (one 100 mg tablet b.i.d. for three days), repeated in subsequent cycles with a 1-month time interval. After the first cycle, a chest X-ray showed that the pleural effusion had improved. Complete recovery was shown after 4 months by X-ray and ecography, being associated to a negative serology result for *T. canis* and to resolution of eosinophilia.

## Conclusions

T. canis infection should be taken into account in cases of pleural effusion resistant to conventional treatment. The *in vitro* detection of *T. canis*-specific IgG antibodies leads to appropriate, effective anti-elminthic treatment.

## Consent

Written informed consent was obtained from the patient for publication of this abstract and any accompanying images. A copy of the written consent is available for review by the Editor of this journal.

